# Hydrogel-Integrated Heart-on-a-Chip Platform for Assessment of Myocardial Ischemia Markers

**DOI:** 10.1021/acsomega.4c02121

**Published:** 2024-09-30

**Authors:** Berna Ates, Tolga Eroglu, Seray Sahsuvar, Ceyhun Ekrem Kirimli, Ozgur Kocaturk, Sahin Senay, Ozgul Gok

**Affiliations:** † Department of Biomedical Engineering, Faculty of Engineering and Natural Sciences, Acibadem Mehmet Ali Aydinlar University, Istanbul 34752, Turkey; ‡ School of Medicine, Acibadem Mehmet Ali Aydinlar University, Istanbul 34752, Turkey; § Department of Medical Biotechnology, Institute of Health Sciences, Acibadem Mehmet Ali Aydinlar University, Istanbul 34752, Turkey; ∥ Institute of Biomedical Engineering, Bogazici University, Istanbul 34684, Turkey; ⊥ Department of Cardiovascular Surgery, School of Medicine, Acibadem Mehmet Ali Aydinlar University, Istanbul 34752, Turkey

## Abstract

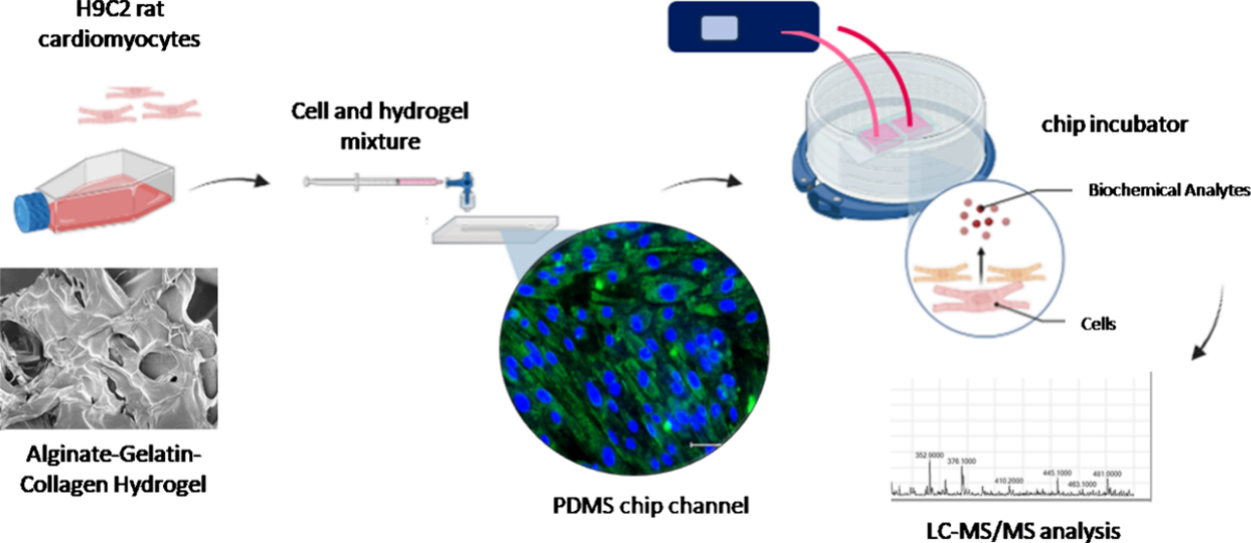

Organ-on-a-chip platform scans offer a controllable environment and a physiological similarity to mimic human pathophysiology. In this study, a single-channel PDMS microchip was fabricated, characterized, and optimized to obtain a heart-on-a-chip platform, which is integrated with a hydrogel scaffold suitable for cardiomyocyte growth inside its channel. Single-channel chips with a size of 20 × 12 mm and a channel height ranging from 60 to 100 μm were produced using photolithography and soft lithography techniques. A gelatin-embedded alginate network-based hydrogel was further augmented with 3% (v/v) collagen type I. Pore sizes were in the range of 74–153 μm for H9C2 implantation and biomimicry. The hydrogels are characterized both on PDMS surfaces and in capillaries. The primary feature distinguishing this study from previous microchip studies is that it mimics the cell microenvironment much better using different hydrogel formulations instead of creating a 2D cell culture by passing fluids, such as fibronectin, for cell adhesion. Instead of using complex microchip designs, the chip system we created intends to provide a physiologically relevant copy by using a 3D cell culture to its advantage and a simple, single-channel architecture. The microchip study was combined with cardiomyocytes to create the heart-on-a-chip system and tested under normoxic and hypoxic conditions to create a myocardial ischemia model inside this channel. As a result, this heart-on-a-chip platform was shown to be utilized for the detection of several small-size biomarkers such as adenosine, ADP, lactic acid, l-isoleucine, l-glutamic acid, and oxidized glutathione via LC-MS/MS from control conditions and a myocardial ischemia model. Cell-embedded and hydrogel matrix-supported versions of this heart-on-a-chip system were successfully prepared and shown to provide powerful outputs with myocardial ischemia markers. In light of this research, these outputs aim to develop simple and biologically effective organ-on-a-chip systems for future research.

## 1 Introduction

Microfluidic chips, where cells are cultured under a medium flow, are an alternative technology to 2D cell culture. “Organ-on-a-chip” technology has been driven by the unique benefits of miniaturizing culture systems, such as increased analytical capacity, improved sensitivity, and analytical performance, and the capability to manipulate and process smaller quantities of reagents.^1^ Recently, “organ-on-a-chip” systems have shown great potential for disease models, drug screening, and toxicity testing.^2^ The literature reveals that cells for in vivo models receive regular chemical and physical stimulations from the surrounding environment, including stretching and shear stresses as well as obtaining oxygen and nutrients through blood flow.^3^ However, in vitro 3D cell culture approaches provide a quasistatic environment in which an analyte evaluation depends on diffusion. Additionally, animal models may not accurately mimic human pathophysiology.^4,5^ For these reasons, organ-on-a-chip platforms have become an emerging research method of interest for in vitro experiments.

Cardiovascular diseases emerge as the primary cause of mortality before age 70 in many countries worldwide.^6^ Myocardial ischemia is a result of coronary heart diseases that eventually might cause myocardial infarction (MI) and sudden cardiac death. Particularly, untreated coronary vascular occlusions have been shown to severely damage cardiomyocytes.^7^ Biochemical markers play a crucial role in early and delayed ischemic preconditioning by showing elevated levels in the serum for myocardial ischemia and infarction (cardiac troponins, CK-MB, adenosine, lactate, etc.) in the clinic. Heart-on-a-chip platforms offer a 3D cardiac tissue microenvironment that mimics natural physiology compared with conventional culture models.^8^ These platforms are designed for developing and screening new drug molecules and investigating disease mechanisms like MI by enabling a more controlled in vitro model for healthy and diseased heart tissue. Various “heart-on-a-chip” designs seem to appear as remarkable approaches that have been elaborately proposed to mimic the dynamic conditions of the cardiovascular system more realistically.^9−11^

3D cell culture environments that utilize hydrogel scaffolds as soft tissue-mimicking platforms have recently gathered a lot of attention and stand out as biologically more advantageous experimental setups compared to 2D ones.^12,13^ With the help of hydrogels, ECM elements can be incorporated inside, such as fibronectin, collagen, and gelatin.^14^ Additionally, their use in microfluidic systems is favorable to obtain better biomimicry as they provide continuous flow conditions experienced by living cells in their in vivo environment, such as cardiomyocytes and endothelial cells. Thus, innovative alternatives, such as the development of in vitro human disease models, are necessary to understand human pathophysiology better.^15−17^

Among the heart-on-a-chip studies, Wang et al. developed 3D heart muscle constructs with beating in vitro in fibrin-based hydrogel media.^18^ Furthermore, the physical cross-linking of alginate chains with Ca^2+^ contributes to the dynamic structure of the resulting hydrogel, thereby supporting the reorganization of cell attachment and migration. Alternatively, entrapment of gelatin chains in cross-linked alginate pores enhances the mechanical properties of the hydrogel by contributing to its integrity with several cell adhesion motives as well as by forming H-bonding.^19,20^ Sarker et al. have found that the diameter of alginate–gelatin microcapsules increased as the swelling of the hydrogel increased and then decreased after 14 days due to the degradation of this polymer mixture.^21^ In a study by Scott et al., a similar trend was observed with different PEG-collagen hydrogels, where increasing the collagen content in the final hydrogel structure resulted in a higher swelling capacity.^22^ Therefore, hydrogel incorporation seems to facilitate a 3D cell culture environment; collagen and gelatin helped in the attachment of the hydrogel to the PDMS surface and promoted cell adhesion within the hydrogel and to the channel surface.^23−26^

Since the mechanical properties of hydrogels play a crucial role in cardiomyocyte orientation, the dynamic conditions (e.g., blood flow under shear stress) for the natural tissue might be modeled for the in vitro cardiac cell culture environment using these microfluidic chip systems.^27^ Kobuszewska and his group investigated the microenvironmental perfusion conditions that improved the cell proliferation and morphology of rat cardiomyoblasts (H9C2) and concluded that the induced parallel alignment is better than static conditions.^28^ Butcher et al. showed that under flow conditions, porcine aortic endothelial cells were aligned parallel to the porcine aortic valve and endothelial cells were arranged perpendicular to the medium flow; however, cells were randomly arranged in a static culture.^15^ In microfluidic chip designs, poly(dimethylsiloxane) (PDMS) surfaces are usually coated with natural ECM components, such as collagen, fibrin, laminin, and gelatin, for enhanced cell adhesion.^29−31^ Ren et al. developed a PDMS microsystem for the dynamic study of hypoxia-induced myocardial injury. The microsystem comprised three microchannels: a central microchannel for H9C2 culture and two side microchannels to control the flow of the culture medium and tested solutions.^32^ Based on these findings, flow and perfusion simulations would appear as an effective tool to improve cell proliferation and arrangement in a single-channel PDMS chip with an integrated hydrogel network. Here, we have successfully fabricated a basic yet effective microfluidic chip with a hydrogel-based 3D network integrated into its channel to mimic the tissue microenvironment better. Favoring cell adhesion by the natural polymer-dependent composition, these hydrogels served as a biocompatible and ECM-like environment for rat cardiomyocytes, encapsulated alive in the channel of this microfluidic chip. Analysis and quantification of MI-associated biochemical markers in normoxic and hypoxic conditions clearly verified this “heart-on-a-chip” system as a novel platform for the early detection of MI-associated biomarkers for future research.

## 2 Materials and Methods

### 2.1 Design and Production of the Microfluidic Chip

The fabrication and lithography steps of the microfluidic chip were conducted based on the literature.^32^ In brief, the design and fabrication steps consist of three stages. First, the model was designed by using computer-aided design software (Autodesk Fusion 360) and printed to a high-resolution photomask for each layer of the chip channels. A silicon wafer (100 mm diameter) was cleaned by rinsing it with acetone. The cleaned silicon wafer was washed with 2-propanol and dried with a nitrogen gun. Then, the dried wafer was placed on a spin coater device, 1 mL of TI Prime was added to the wafer, and the coating was set by using the following spin program: ramp up to 500 at 100 rpm/s acceleration for 1 s, and 4000 at 300 rpm/s acceleration for 30 s. The coated wafer was baked on the hot plate at 95 °C for 2 min. The heated wafer was placed on the top of the spin coater device, and 4 mL of SU-8 2050 negative photoresist was added to the wafer. The spin-coating process was set by the following spin program: ramp up to 500 at 100 rpm/s acceleration for 10 s, 1900 at 300 rpm/s acceleration for 30 s, and finally 2900 at 2900 rpm/s acceleration for 1 s. The coated wafer was baked at 65 °C for 5 min and 95 °C for 20 min and then cooled at 65 °C for 2 min. Afterward, the printed high-resolution photomask was placed into the mask positioner and aligned with the wafer providing a 25 μm gap and exposed to UV light. After UV light exposure, the wafer was heated successively at 65 °C for 2 min, at 95 °C for 10 min, and at 65 °C for 1 min. The lithographed wafer was placed in a 120 mm Petri dish, and the SU-8 2050 developer was poured onto it to obtain a thickness of approximately 60–100 μm. Next, the Petri dish was gently shaken for 11 min to eliminate the non-UV-exposed SU-8 2050 negative photoresist. The wafer was rinsed with isopropyl alcohol and dried using nitrogen gas. While the channel protuberances were produced on the wafer facing up, 100 μL of silane was poured over its surface, and the wafer was placed in an oven to evaporate the silane at 65 °C for 1 h. The second step consists of a soft lithography process. In a separate beaker, 50 g of PDMS and 5 g of curing agent (10:1 (v/v) ratio) were mixed, and the homogeneous mixture was left under vacuum for 1 h to remove bubbles. The PDMS mixture was evenly poured on the wafer and left in the oven at 65 °C for 3 h for curing after the silane had evaporated. The third step comprises the device assembly. The cured PDMS chip layers were removed from the wafer by using a blade. The inlets and outlets of the culture channels were drilled using a 2 mm diameter biopsy punch, and the bottom layer of the channel was also punched to facilitate gas diffusion. The upper and lower layers of the chips underwent oxygen plasma treatment for 3 min to eliminate the surface contaminants. Then, a membrane was positioned between the chip layers and was compressed together for 10 s. To improve the adhesion, the chips were placed in an oven at 65 °C for 24 h.

### 2.2 Hydrogel Optimization, Characterization, and Adhesion Studies

#### 2.2.1 Hydrogel Preparation

A cell-compatible hydrogel is needed for cardiomyocytes to attach inside the PDMS channel and grow into a 3D structure. A hydrogel mixture was optimized with different concentrations of 0.50% (w/v) gelatin, 0.50% (w/v) alginate, and 0.55% (w/v) CaCl_2_ for the alginate cross-linking and incubated at room temperature for 20 min for complete cross-linking. These solutions were prepared in double-distilled water (ddH_2_O). The obtained gelatin–alginate hydrogels were designated as “HG”.

#### 2.2.2 Hydrogel Adhesion to Glass Capillaries

The hydrogel optimized in the previous experimental step was created by adding gelatin, alginate, and CaCl_2_ solutions sequentially to a 1 mL syringe. After initiating cross-linking with calcium, the hydrogels were homogeneously mixed. The hydrogel solution was quickly transferred to a glass capillary, and these steps were repeated four more times in four different capillaries and in a separate beaker, and then all were placed in an orbital shaker. At 0, 10, 20, 30, and 40 min, 1 mL of ddH_2_O was injected twice into the capillaries. The gelation state in the beaker was observed and compared simultaneously.

#### 2.2.3 Determination of Collagen Concentration and Hydrogel Swelling Studies

To improve the adhesion of the hydrogel to the surfaces, various amounts of collagen solution (1, 2, 3, and 4% (v/v)) were added separately to 2 mL of gelatin–alginate hydrogel mixtures to obtain HG-C1 to HG-C4, separately, before cross-linking. After complete gelation, the hydrogels were dried by lyophilization. Dried gels were weighted, and known amounts were incubated in excess ddH_2_O for time-dependent swelling. A series of weight measurements were taken at different time points (up to 62 min) for each hydrogel formulation. The increase in protein content corresponding to the amount of collagen in the HG-C 1–4 hydrogels was analyzed by the BCA assay (Thermoscientific Pierce BCA Protein Assay Kit). The BCA assay procedures were performed according to the instructions provided in the kit’s manual.

#### 2.2.4 Short-Term Hydrogel Degradation Study

The integrity of the hydrogel was assessed by weight measurements at various time points for the hydrogel HG-C3 incubated in ddH_2_O for up to 7 days at 37 °C. The short-term stability of the hydrogel was assessed along with its swelling profile.

#### 2.2.5 Morphological Analysis

The porous structure of HG-C3 was evaluated using a scanning electron microscope (SEM, Leica Quanta 650 FEG). The lyophilized HG-C3 was cut into four quarter pieces, each containing both the contact surfaces as well as the inner and upper surfaces. Under SEM, the cutting surface showed a porous internal architecture. While analyzing the glass contacting and top surfaces, the surface morphology and pore diameter were assessed at magnifications ranging from 35× to 20,000×.

#### 2.2.6 Structural Characterization

The chemical structures and characteristic functional groups of the polymers present in the hydrogel structures were analyzed on a lyophilized HG-C3 hydrogel using Fourier transform infrared (FT-IR) spectroscopy (Thermoscientific Nicolet iS10).

#### 2.2.7 Mechanical Analysis

At room temperature, the hardness and flexibility properties of the HG-C3 hydrogel were measured by using a rheometer device (Malvern Kinexus+) at a frequency of 1 Hz.

### 2.3 Optimization of the Combination Time of Collagen with the Hydrogel

The adhesion of polymer solutions to collagen-coated surfaces at various time points was an important variable that affected the adhesion strength of the HG-C3 hydrogel. Thus, four different sets of experiments were designated on glass slides (named GS_) and PDMS surfaces. For the procedures applied to the surfaces, refer to supp. data Appendix 1. PDMS capillaries were produced to mimic the microchip channel, and the production process can be found in Appendix 2 and Figure S3. Similarly, in the PDMS-c2 and PDMS-c3 named PDMS capillaries, GS2 and GS3 procedures were used, respectively, by injecting the gelatin and alginate solutions along with the collagen solution. The experimental steps to produce the PDMS capillary (PDMS-c) are described in Supp. data (Appendix 2).

PDMS-c2: 20 μL of collagen was injected into the PDMS capillary and left for 15 min. Then, the hydrogel HG was transferred to the channel and left for 30 min.

PDMS-c3: The HG-C3 hydrogel, prepared similarly for glass capillaries, was injected into the PDMS capillary and incubated for 30 min.

#### 2.3.1 Contact-Angle Measurements

The wettability features of glass, PDMS, and collagen-hydrogel-coated PDMS surfaces were assessed using a contact-angle instrument (Attension Theta Lite Optical Tensiometer (Biolin Scientific, Sweden/Finland)) with 4 μL of distilled water.

### 2.4 Preparation of Cell-Loaded Hydrogels

H9C2 rat cardiomyoblasts were obtained from the ATCC and cultured in a complete medium consisting of Dulbecco’s modified Eagle's media with 10% fetal bovine serum. The cells were maintained at 37 °C in a humid incubator with 21% w/v O_2_ and 5% w/v CO_2_. Upon reaching 80% confluency, the cells were detached using 0.25% trypsin. Following detachment, the cells were mixed with hydrogel components prepared with DMEM and without CaCl_2_. Subsequently, CaCl_2_ was added for alginate cross-linking. The final cell density was adjusted to 1 × 10^5^ cells/mL. A 1 mL portion of the polymer–cell mixture was placed onto a 60 mm Petri dish, followed by the addition of 3 mL of complete medium to maintain cell viability. The Petri dish was then incubated in a humidified incubator for 72 h to allow for cell growth.

After 24 and 72 h of incubation, the waste medium was discarded, and cell-hydrogel constructs were washed three times with PBS. A 4% paraformaldehyde solution was added to fixate the cells at 36 °C for 15 min. The fixated samples were stained with DAPI (Thermo Fisher) and AlexaFluor488 (Concanavalin A, Thermo Fisher) fluorescence dyes as per the manufacturer’s instructions. Morphological changes in cardiomyocytes were visualized using a fluorescence microscope (EVOS Cell Imaging System).

### 2.5 Pressure-Controlled Microfluidic Flow

A pressure-controlled microfluidic flow system (Elveflow, Paris) was used to integrate the prepared hydrogel mixture into the channel of the microfluidic chip. A 3 mL HG-C3 hydrogel mixture was prepared within a 5 mL syringe. After cross-linking was completed, the syringe-chip adapter was used to transfer the hydrogel from the syringe into the PDMS chip in a controlled manner. The PDMS chip with the hydrogel was incubated at 37 °C for 1 h to promote the adhesion of the hydrogel. The hydrogel formation was verified by injecting 0.1% methylene blue and observing its passage through the channel. For the passage of biomarker solution, different concentrations (0.75, 1, and 1.5 μM) of 20 mL of adenosine solutions were prepared and transferred to the culture channel at 40 mbar. The channel was rinsed with 2 mL of ddH_2_O between each injection. In each experiment, midstream incoming solutions were collected and stored at −20 °C for further analysis. The LC–MS/MS (Agilent Triple Quad 1640) system was used to quantitatively assess the incoming and outgoing adenosine solutions for concentration changes. A C18 column was used as the stationary phase, and 50%ACN:50%H_2_O was passed through it at a rate of 0.5 mL/min as the mobile phase. The multiple reaction monitoring method for the pure adenosine molecular ion was set at 268.1 g mol^–1^ (theoretical *M*_wt_: 267.24 g mol^–1^) and is used to obtain the calibration curve.

### 2.6 Cardiomyocyte-Loaded Hydrogel Integration into the Chip

The effect of normoxic and hypoxic conditions on hydrogel-encapsulated cells was determined by an experimental setup including cells seeded into six-well plates with a final cell concentration of 1 × 10^5^ cells/mL. The well plates were incubated in a normoxic incubator for 24 h to allow the cells to adapt to the hydrogel. After incubation, the medium was changed and 0.5 mL of medium samples were taken at the end of 1, 6, 24, and 48 h. In the hypoxia group, after 24 h of incubation, the well plate was placed in a mini-incubator. The gas mixer of the mini-incubator was adjusted to 5% CO_2_, 94% N_2_, and 1% O_2_, and 0.5 mL of medium samples were taken at the end of 1, 6, 24, and 48 h after the hypoxia conditions started. Collected samples were stored at −20 °C.

The chip channels were sterilized with 70% (v/v) ethanol and UV exposure for 1 h before the channels were incubated with a collagen type I coating solution for 24 h in a cell incubator. The channels were washed with sterile PBS, and the hydrogel components were transferred into the syringe, followed by cell solution transfer into the same syringe at a final concentration of 1 × 10^5^ cells/mL. After complete cross-linking and gelation, the excess fluid that accumulated in the syringe was removed from the syringe. Then, the actual hydrogel-cell mixture remaining in the injector was transferred to the chip channel through a syringe-to-chip adapter. The chip was left in the mini-incubator (Ibidi Stage Top Incubation System) for 4 h for complete gelation and cellular adhesion. Upon attachment, the chip was connected to the microfluidic flow device (Elveflow), with a mean flow rate of 60 μL/h and mean and maximum pressures of 40 and 60 mbar, respectively. The two ends of the chip were then connected to the microfluidic flow system to perfuse the cells with a fresh medium, and the chip platform was placed in the closed mini-incubator overnight. The temperature on the floor plate and inside the incubator was set to 37 °C. Following incubation, the chips were perfused in the incubator for 48 h under normoxic and hypoxia conditions. To establish the hypoxic conditions, the cell medium was kept under a gas mixture of 5% CO_2_, 94% N_2_, and 1% O_2_ for 24 h before the chip perfusion. At 1, 6, 24, and 48 h, medium samples were withdrawn from the chip outlets under both normoxic and hypoxic conditions and kept at −20 °C. Collected samples were analyzed by an LC-MS/MS instrument for small-molecule detection and adenosine quantification. The cells inside the chips were fixated with 4% paraformaldehyde and stained with DAPI (for cell nuclei) and Conc.A-AlexaFluor488 (for cell membranes). Chip cross sections were visualized with the fluorescence microscope.

## 3 Results and Discussion

The presented study involves the design and characterization of a hydrogel-integrated heart-on-a-chip platform with promising features of chip-based microfluidic systems, which is associated with a 3D microenvironment for cell cultivation to mimic an in vitro myocardial ischemia model.

### 3.1 Design and Production of the Microfluidic Chip

**Figure 1:**
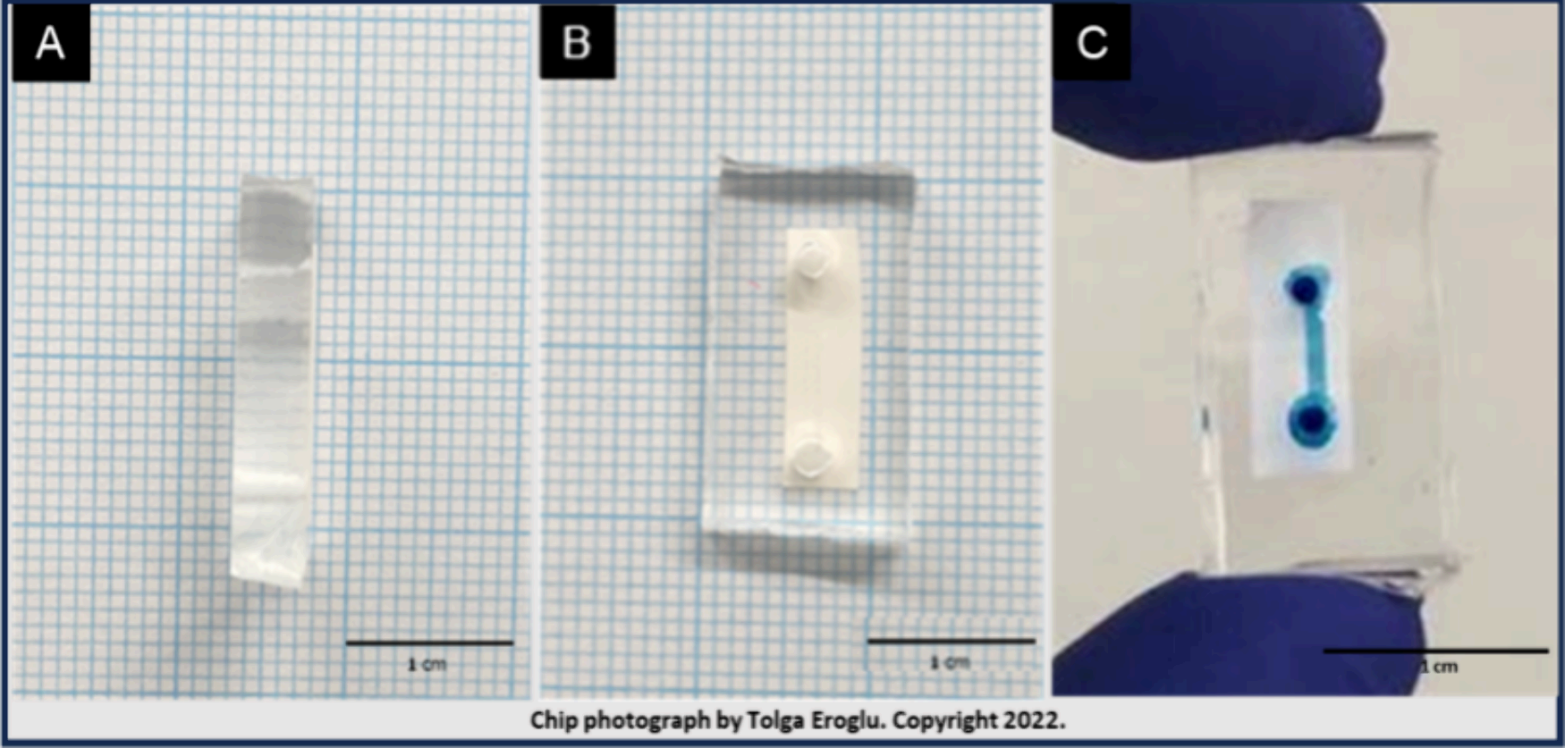
(A, B) Combined PDMS layers after oxygen plasma treatment. The combined layers formed the 20 mm × 12 mm × 3 mm PDMS chip. (C) Result of the water tightness test conducted with methylene blue.

The chip platform was fabricated using the photolithography technique in combination with the soft lithography technique to obtain the PDMS molds for microchannel patterns along with heights ranging from 60 to 100 μm on the wafer. Photolithography-based chip fabrication was used to obtain a single-channel microchip. After curing for 24 h, the prepared chips were removed from the oven, and a 0.1% (w/v) methylene blue solution was passed through the chip channel for the liquid leakage test. Figure 1 illustrates the layers of the bound chip after the liquid leakage test, demonstrating that the chips are watertight and are eligible for cell culture. Figure S1 illustrates the wafer and uncut chip layers. One remarkable feature of this chip design is the hole punched at its bottom for contact with the air inside the culture chamber through the membrane. The nonleaky structure of these holes was tested using methylene blue solution. We managed to achieve successful bonding of PDMS layers to fabricate these highly elastic microfluidic chips through a thorough cleaning of their surface from contaminants with oxygen plasma (Figure 1).^33^

### 3.2 Hydrogel Optimization, Characterization, and Adhesion Studies

As cross-linked polymeric networks, hydrogels provide a porous environment for encapsulation of cells, and their composition has a direct effect on the adherence of these living cells to polymeric walls inside. The cardiomyocytes were implanted in the prepared hydrogel structure, which serves as a 3D environment for their implantation and adherence. The attachment tendency of implanted cardiomyocytes was controlled by both the composition of this hydrogel scaffold and its cross-linking density. In this study, a physically cross-linked alginate polymer was utilized for the fabrication of the hydrogel, and this network was reinforced by the interpenetration of natural ECM-mimicking biocompatible polymers like gelatin and collagen type I.

**Figure 2:**
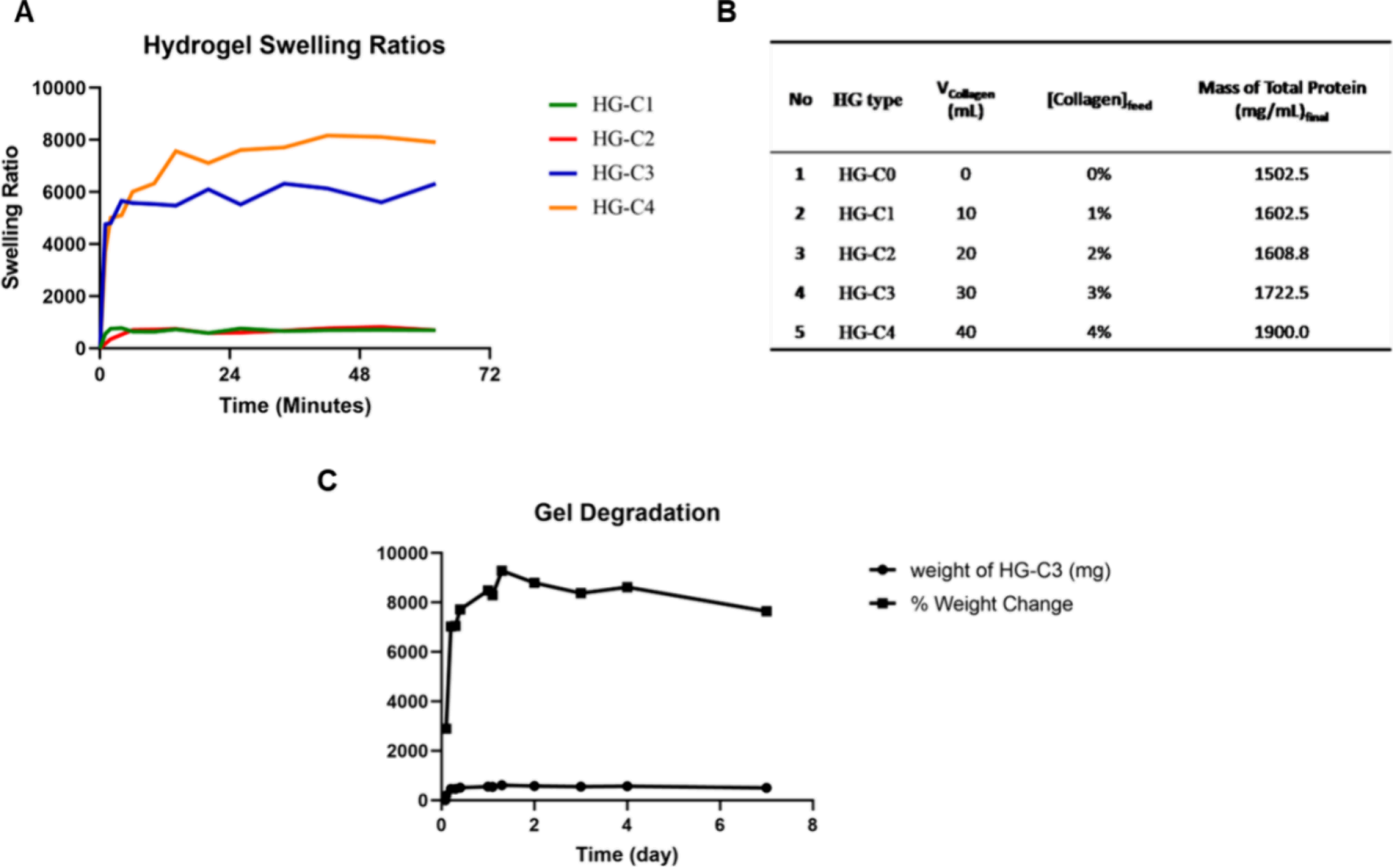
(A) Comparison of water uptake capacities for HG C1–4. (B) Results of the BCA protein assay for HG C1–4. (C) HG-C3 degradation profiles were obtained in ddH_2_O at 37 °C for 7 days.

A stable hydrogel scaffold structure was achieved by combining calcium cross-linked alginate with free gelatin chains. Among the five glass capillaries kept at different incubation periods, hydrogel adhesion was observed after 20 min. Adding collagen solution to the hydrogel improved the adhesion to the glass surface. Hydrogel swelling profiles, as shown in Figure 2A, compare the hydrogels with varying collagen contents. The hydrogel with 3% collagen by volume (HG-C3) was chosen for subsequent steps due to its favorable swelling characteristic and strong adhesion feature.
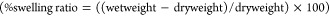
1

Additionally, the collagen coating was found to increase the contact angle of the PDMS surface, thereby promoting hydrogel adhesion. A time-dependent investigation of this process revealed that a complete hydrogel adhesion occurred at the 15th min of incubation after the spin-coating step.

Results for the protein quantification experiments are demonstrated for the hydrogels with varying collagen concentrations in Figure 2B. The findings indicate a direct correlation between the total protein and the increase in collagen content of the obtained hydrogels. The HG-C3 hydrogel weight was observed to swell continuously for up to 2 days at 37 °C. Fluctuations in the weight afterward can be attributed to hydrogel degradation; however, it did not lead to significant loss in the mass for up to 7 days (Figure 2C).

**Figure 3:**
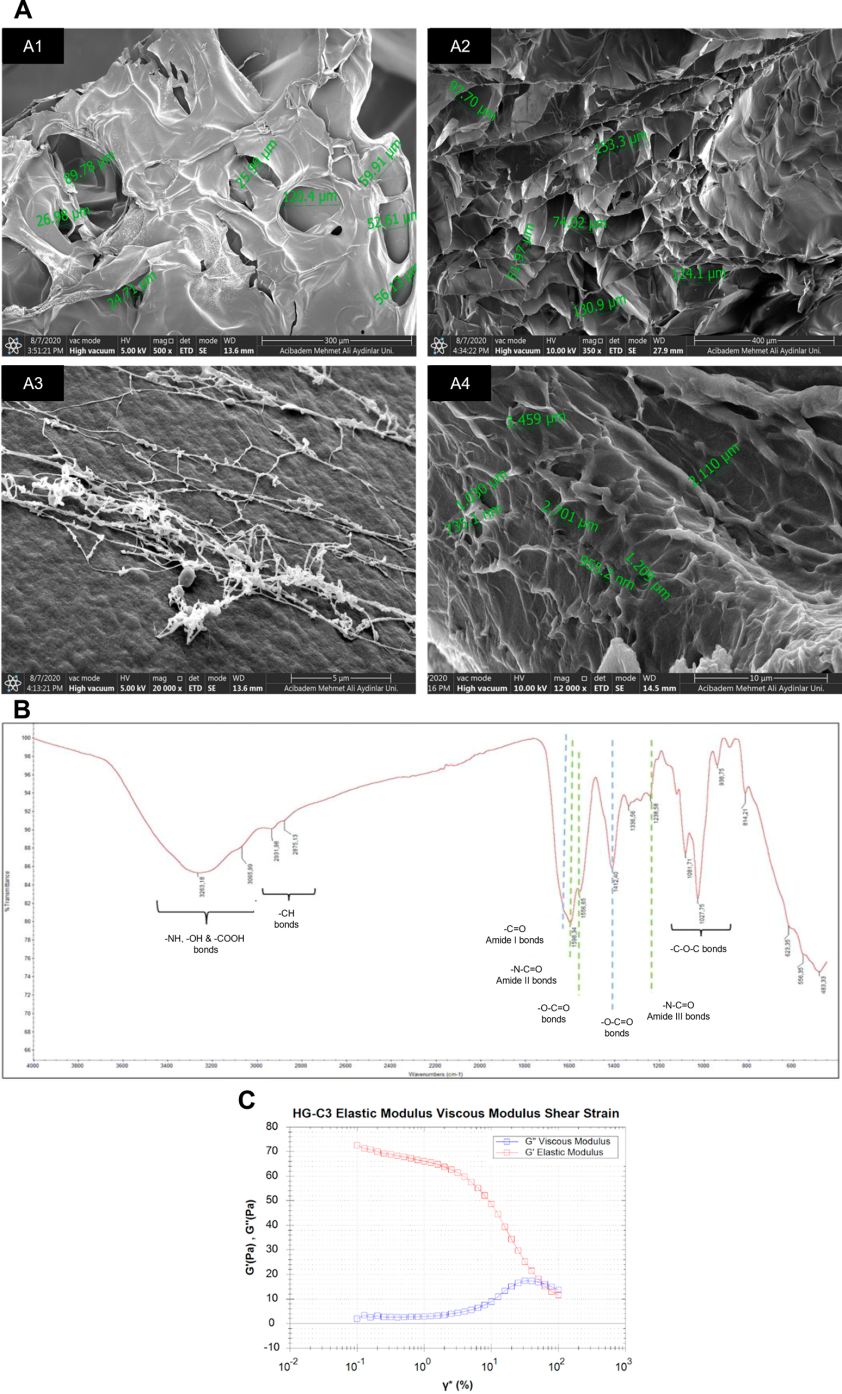
Morphological characterization of HG-C3 via SEM under low vacuo. (A1) A porous structure can be seen (pore widths vary in the range of 74–153 μm) in the top view of the HG-C3 hydrogel, and salt formation due to excess ions (CaPO_3_) is distinctly seen at 500× magnification. (A2) Cross-sectional view at 350× magnification, displaying the porous structure and density of the pores. (A3–4) Reticulated collagenous structures at 20,000× and 12,000× magnifications, respectively. (B) FT-IR spectrum of the HG-C3 hydrogel. The amine and carboxylic acid groups from the chemical structure of the gelatin and characteristic carbonyl peaks arising from alginate, gelatin, and collagen polymer are observed as expected. (C) Viscoelastic behavior of the HG-C3 hydrogel.

The HG-C3 was evaluated with SEM for its surface morphology and porous structure at different magnifications (35, 100, 1000, and 2000×), as illustrated in Figure 3A. The overall hydrogel morphology was analyzed at low magnification, where the external porous surface separation was better distinguished at higher magnifications. The cross-sectional view revealed detailed information about the pore structure with diameters ranging from 74 to 153 μm, which is suitable for future cardiomyocyte implantation.^34^ Also, the impact of collagen addition into the hydrogel scaffold was demonstrated as the reticulated fibrillar structure that ensured its cell adhesive feature at high magnifications.^35^ These reticulated structures were observed at 1000× on surfaces in contact with glass, and they were determined to be collagen fibrils at 20,000×.

The chemical structure of the HG-C3 hydrogel was confirmed using FT-IR spectroscopy, and Figure 3B shows the obtained spectrum. The stretching bands corresponding to the amine and carboxylic acid groups in the chemical structure of the gelatin polymer were observed as a wide band in the frequency range of 3300–3500 cm^–1^, as expected. Furthermore, the characteristic carbonyl peaks originating from the chemical structure of alginate and the gelatin polymer as well as collagen were observed at frequencies between 1642 and 1412 cm^–1^. Furthermore, the mechanical properties of this hydrogel scaffold were investigated using the oscillatory stress sweep test at *f* = 1 Hz and with γ = 0.01, at 25 °C. According to this rheometer analysis of the HG-C3, upon increasing pressure, a drop in the *G*′ representing the solid (elastic) part of the gel and an increase in the *G*′ representing the liquid (viscous) part were observed (Figure 3C). This contributes to an increased viscoelastic behavior of the hydrogel caused by the resultant deformation, which lasts up to almost γ = 100 at the cross point.

**Figure 4:**
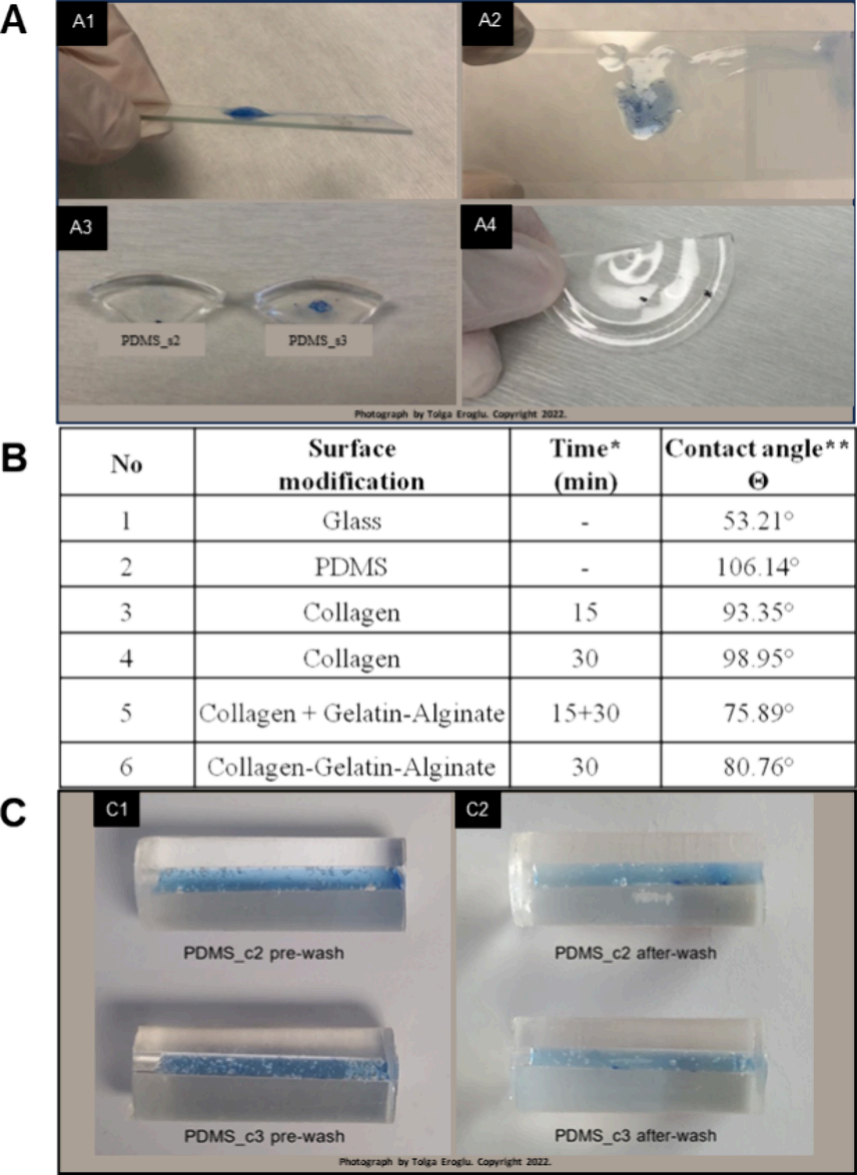
Side (A1) and top (A2) views of HG-C3 on a glass slide. PDMS surface coated with collagen only (A3) and HG-C3 adhered on it (A4). (B) Contact-angle measurement results. Hydrogel retention in PDMS capillaries before (C1) and after (C2) water washing steps for PDMS-c2 and PDMS-c3. *Time values represent the incubation time of hydrogels on the surface. **For all measurements, the volume of the water droplet was set to 4 μL.

The prepared hydrogel needs to be attached to the chip materials to provide a stable environment for living cells. In our study, after optimization of the hydrogel composition, the prepared hydrogels were characterized for their stability in the chip channel. We introduced collagen into our hydrogel network to improve cell adhesion. For this purpose, the hydrogel mixture was investigated for its adhesion tendency toward both glass and PDMS surfaces with or without collagen addition. However, no difference was observed in the water flow resistance test between collagen precoated or noncoated PDMS capillaries. These results overall point out the proper adhesion of collagen, including hydrogels, with the PDMS surface of the microfluidic channel without causing any decrease in the flow rate through the channel. Figure 4A,C shows the related PDMS hydrogel adhesion results. For the glass surface, stronger hydrogel adhesion was observed on the GS2 slide compared with that on the GS3 slide, whereas the hydrogels on GS1 and GS4 surfaces were easily removed from the glass slides (Figure 4A). The same result was reached in PDMS surfaces (PDMS-s_), PDMS-s2 and PDMS-s3, where the GS2 and GS3 procedures were applied in the same way. The procedures applied to the surfaces were replicated in the produced PDMS capillaries. Pure ddH_2_O was passed through the hydrogel-loaded PDMS capillaries (PDMS-c2 and PDMS-c3) by using an injector to ensure the adhesion of the hydrogel. After water flow, both hydrogels were found to be attached to the capillary surfaces (Figure 4C). Thus, hydrogel injection procedures were confirmed to be suitable for its implantation into PDMS chip channels.

The wettability properties of surfaces and contact-angle values against water were measured and assessed for the hydrogel contents. As expected, the PDMS surface had a higher contact-angle value (106.14°) than the glass surface (53.21°), confirming the hydrophobic nature of PDMS. Coating the PDMS surface with a hydrogel solution (gelatin–alginate mixture) with or without collagen was observed to decrease the contact-angle value, as demonstrated in Figure 4B. The contact-angle value increased from 93.35° (at 15 min) to 98.95° (at 30 min) due to an increase in the spin-coating time for the PDMS surface.

In Figure 4B, the addition of a gelatin–alginate mixture to the collagen-coated surface gives a contact-angle value of 75.89° at its 15th min, while the value increased to 80.76° when gelatin–alginate and collagen were added to the surface simultaneously and spin-coated for 30 min. In conclusion, collagen coating for 15 min on the PDMS surface offered a more hydrophilic surface for better hydrogel adhesion.

### 3.3 Pressure-Controlled Microfluidic Flow Estimation

Integrating this hydrogel into the channel and flow of an aqueous solution through body pressure using a pressure-controlled microfluidic flow system was an important step that determined the suitability of this chip to be used as an in vitro model. Adenosine, a biomarker that increases during MI, was evaluated using this system. Different concentrations of this biomarker were passed through the channel of the chip with a driving pressure of 40 mbar, which corresponds to the mean capillary pressure. Recovery percentages increased with the concentration of the adenosine solution, and this relation is essential for monitoring the detection of released adenosine during a flow-based dynamic environment under ischemic stress.

**Figure 5:**
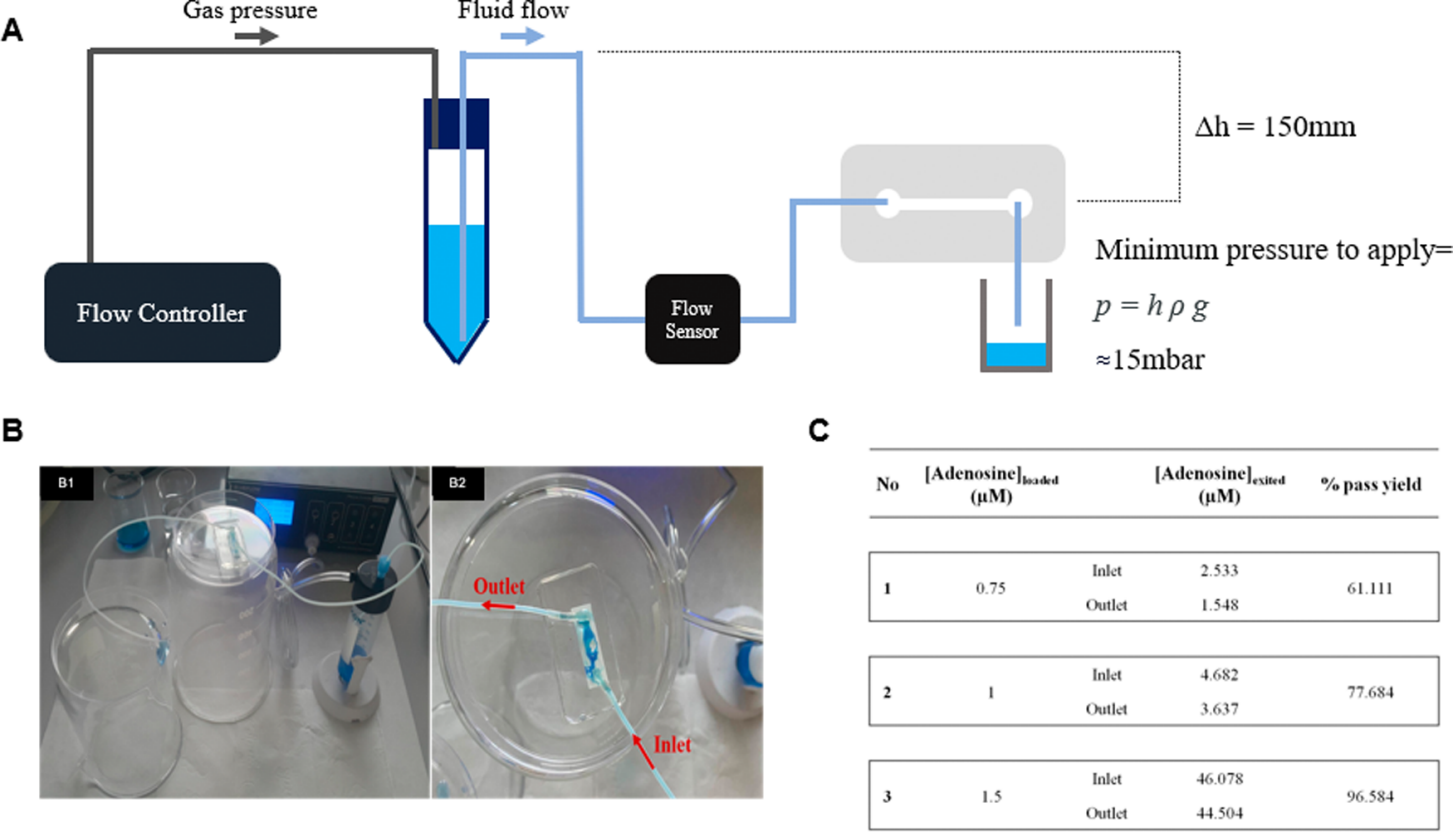
(A and B1) Pressure-controlled microfluidic system setup for the microfluidic chip. (B2) Image of the adhered hydrogel inside the chip channel. (C) Concentrations and recovery yields of adenosine solutions passed through the microfluidic chip system.

During the microfluidic flow experiment, the concentrations of all adenosine solutions (0.75, 1, and 1.5 μM) were detected to be decreased after passing through the hydrogel inside the channel. Figure 5A,B shows the pressure-controlled microfluidic system setup.

The lowest recovery percentage for this passage, calculated as the ratio of parts per billion of the outlet solution to parts per billion of the inlet solution, was found to be 61.11% for the 0.5 μM feed adenosine solution. The recovery yield of adenosine was found to be increased along with that of the initial solutions. The highest recovery yield of 96.58% was obtained for the adenosine solution, with an initial concentration of 1.5 μM. Figure 5C presents the adenosine concentrations of the inlet (initial) solutions and the outlet (eluted) solutions, determined by an LC–MS/MS instrument.

### 3.4 Cell-Loaded Hydrogels

**Figure 6:**
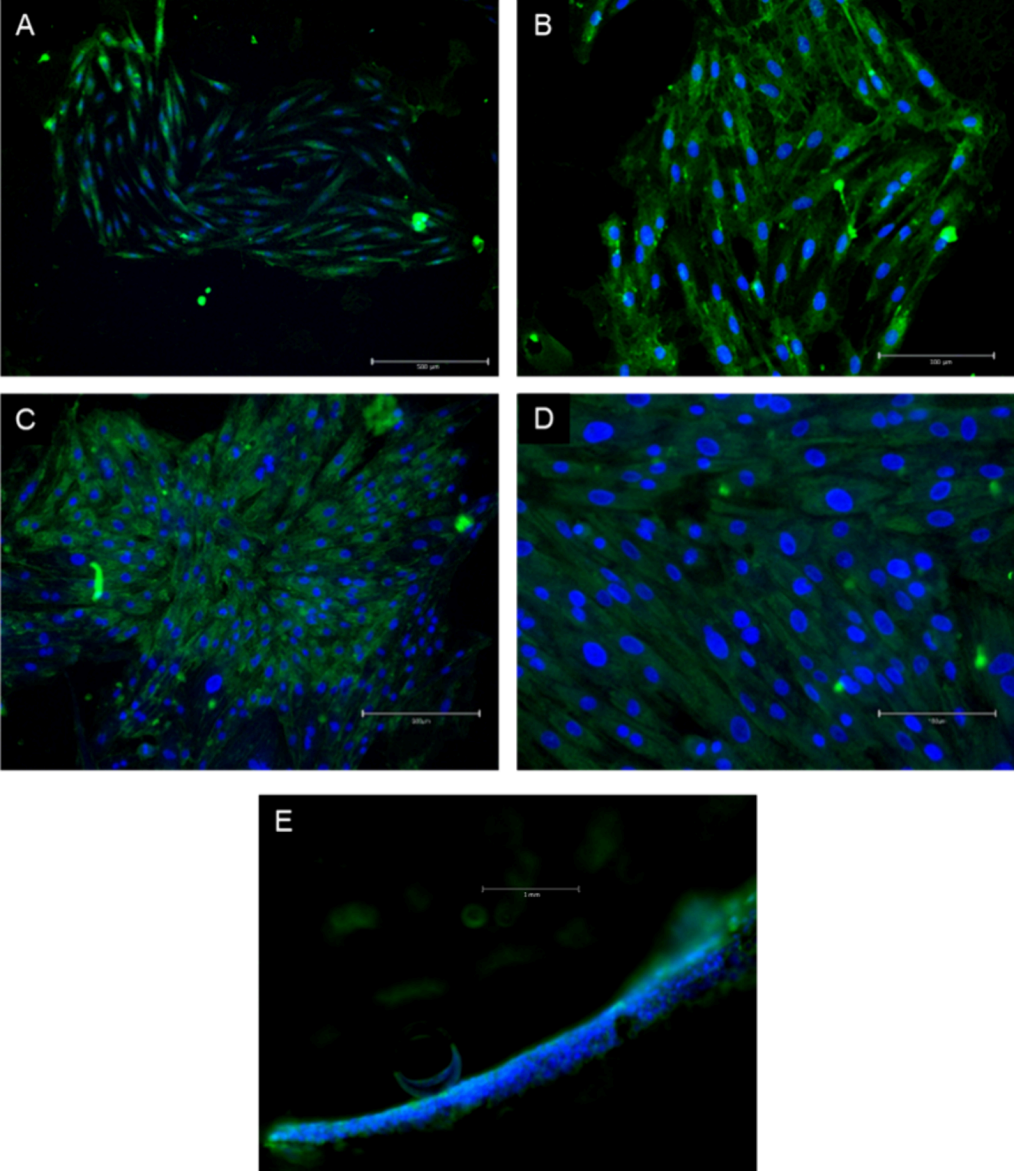
Fluorescent microscopy images of H9C2 cells grown in the HG-C3 hydrogel in a microfluidic chip channel, fixed at 24 h (A, B) and 72 h (C, D), and stained with DAPI (blue) and AlexaFluor488 (green) (scale bar: 500 μm for A and C, 50 μm for B and D). (E) Cross-sectional view of the chip channel after 72 h of normoxic perfusion. Staining with DAPI and AlexaFluor488 shows cardiomyocyte adhesion on the channel surface.

The hydrogel was mixed thoroughly with the cell suspension to create a homogeneous polymer–cell mixture, which was later on injected into the channel of the prepared microchip. Cardiomyocytes were retained inside the channel with the help of the prepared hydrogel scaffold inside by being entrapped in its pores. After the incubation of these cells in the microchip under sterile conditions and at 37 °C, the hydrogel-integrated cardiomyocytes were incubated for 24 and 72 h under normoxic conditions. At the end of 24 h, fluorescence imaging of the cells with DAPI and AlexaFluor488 (Concavalin A linked) showed that the cells were able to adhere to the hydrogel and form connections with each other (Figure 6).

At 72 h of incubation, both the cell proliferation level and the green fluorescent signal in the cytoplasm were observed to be increased. Concanavalin A binds to intracellular conjugated glycoproteins, meaning that intracellular glycoprotein synthesis increased over 72 h. As a result of these two findings, it was concluded that the resulting hydrogel forms a cell-compatible scaffold for living cells and, at the same time, has no adverse effect on cell growth and proliferation.

**Figure 7:**
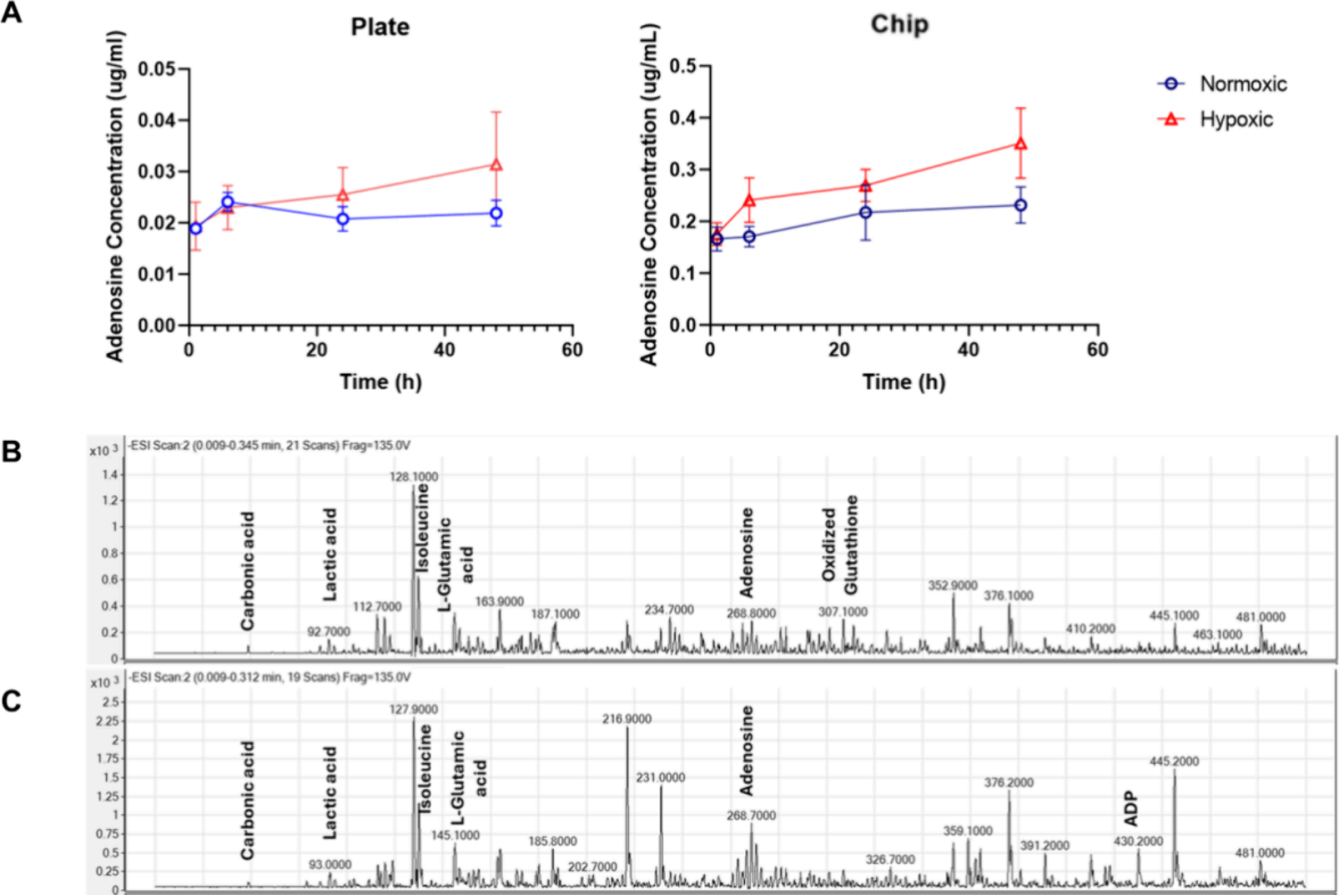
(A) Time-dependent change of adenosine concentration in media samples, obtained from hydrogel-integrated cells in the plate or chip under normoxic and hypoxic conditions. (B, C) LC-MS/MS analysis of cell media obtained from the heart-on-a-chip system after 24 h. Mass spectra for the collected samples were obtained under normoxic (B) and (C) hypoxic conditions.

The hydrogel-integrated cardiomyocytes were exposed to normoxic and hypoxic conditions on a six-well plate, and adenosine concentrations were determined in medium samples collected at certain time points (Figure 7A). At 1 h, the adenosine concentration under normoxic and hypoxic conditions was almost the same at 0.020 μg/mL, whereas at 6 h, it increased in both groups up to 0.024 μg/mL. However, at 24 h, the adenosine concentration in the hypoxic condition continued to increase to 0.026 μg/mL, while it decreased back to 0.20 μg/mL for the normoxic condition. In other words, the first ischemia-induced adenosine difference started to occur at 24 h of incubation. On the other hand, at 48 h, this difference became more noticeable, and the adenosine concentration for the hypoxic condition increased to 0.030 μg/mL, whereas it remained at the same level as at 24 h in the normoxic group.

The analysis of the medium samples collected for both normoxic and hypoxic conditions revealed that the adenosine concentration in the medium samples from the chip was almost 10 times more concentrated than the concentration of the medium collected from the plates (Figure 7A). The finding seems to confirm the efficiency of the designed “heart-on-a-chip” system in delivering higher analyte concentrations at lower volumes. Normoxic and hypoxic cells gave an adenosine concentration of 0.18 μg/mL at the end of the first hour. It increased moderately to 0.20 μg/mL in the normoxic group at the sixth hour, whereas in hypoxic conditions, unlike the behavior in the plate, this concentration was increased to 0.24 μg/mL. These changes suggest that the chip conditions responded much more rapidly than the plate conditions for the cells under hypoxia stress. At the end of 24 and 48 h of incubation, this difference continued to become higher, and at 48 h, the concentration in normoxic conditions remained at 0.20 μg/mL, whereas in hypoxic conditions, it almost doubled to 0.35 μg/mL. Compared to the clinical settings, earlier reports show that the adenosine concentration was found to be 1 μM in the coronary sinus during the myocardial ischemic process.^36^ Thus, the consistent result obtained in this experiment clearly indicates that our “heart-on-a-chip” platform was able to successfully demonstrate the increase in the amount of adenosine secreted by cardiomyocytes into the medium as a result of myocardial ischemia conditions by providing a suitable myocardial ischemia model for in vitro conditions.

Other than adenosine, metabolic intermediates like succinate can also build up during ischemia, resulting in the formation of reactive oxygen species (ROS) after reperfusion as stated in the Chouchani et al.’s chip system.^37^ Moreover, Ren et al. used carbonyl cyanide-*p*-trifluoromethoxyphenylhydrazone, a frequently used chemical hypoxia reagent that has the ability to separate the respiratory chain of the mitochondria, requiring glycolysis for energy production. They monitored the caspase-3 activation signal as the result of myocardial ischemia-apoptosis induction.^16^ The heart-on-a-chip created by Liu et al. simulates nonuniform oxygen distribution, which allows for the investigation of the myocardial hypoxia’s electrophysiological response. For a total of 5 h, they exposed HL-1 cells to a hypoxic medium (1% O_2_). Immunostaining and electrophysical analyses showed that HIF-1α was highly expressed and had become localized in the nucleus within 2.5 h.^38^ With the use of a gas microexchanger and a heart-on-a-chip, Elvassore et al. found that hypoxia could cause a reversible change in the Ca^2+^ concentration in cardiomyocytes. This allowed for in-line analysis of the intracellular calcium concentration using confocal microscopy on neonatal rat cardiomyocytes.^39^ Veldhuizen et al. presented a model of myocardial ischemia on a chip using stem-cell-derived cardiomyocytes and cardiac fibroblasts within a collagen-based hydrogel platform. They reported the relation among the ischemia reperfusion injury, tissue fibrosis, and lactate level re-establishment along with molecular-level analyses.^40^ By exposing the cells to intervals of hypoxia lasting 3–4 h, followed by periods of normoxia, the chip from Khanal et al. was shown to maintain cardiomyocyte culture and induce I/R damage.^10^

In the heart-on-a-chip system, changes in small molecules were normally found within 24 h from normoxic to hypoxic conditions. These findings present the necessity of an analysis of small molecules that change consistent with the myocardial ischemia level. In our study, the collected samples were analyzed by an LC-MS/MS instrument by the injection of the same sample volume so that the relative levels of some myocardial ischemia markers could be compared with each other for both normoxia and hypoxia conditions (Figure 7B,C). Results revealed that no significant change was found in the carbonic acid levels. Heart cells are metabolically active cells and normally use oxygen for energy production.^41^ This process can result in the formation of CO_2_ and the release of carbonic acid (H_2_CO_3_) when carbon dioxide reacts with H_2_O. However, carbonic acid production in heart cells is not as expected compared to other cell types and usually occurs as a byproduct of metabolic processes. Although tissue damage and changes in metabolic activity can impact the synthesis of carbonic acid in circumstances like heart failure, there has not been any stated clear correlation between carbonic acid production in heart cells and this damage.^42^

While a significant increase was observed in the adenosine diphosphate (ADP) molecule under hypoxic conditions, there was also an approximately 0.5-fold increase in the lactic acid levels. During MI, aerobic metabolism is disrupted, as the heart muscle is suddenly deprived of oxygen to meet the energy requirements of the cells. With the increase in anaerobic metabolism, the accumulation of byproducts such as lactic acid increases, which can cause low pH and cellular damage.^43^ This may lead to the release of metabolites such as ADP. In addition, in cases of oxygen deficiency, while adenosine triphosphate (ATP) production decreases due to the incomplete breakdown of glucose, cells can increase the conversion of ATP to ADP and subsequently to adenosine monophosphate (AMP) derived from the adenine base.^44,45^ In this case, increased levels of lactic acid and ADP may be associated with the diagnosis or severity of MI.^46^

The remarkable change seen in the hypoxic experimental setup is for the adenosine molecule, with an approximately 2-fold increase. Ischemia affects the metabolism of energy sources, such as ATP, for which the breakdown is increased during ischemia. This can lead to a direct increase in adenosine levels.^47,48^ Additionally, adenosine is released by cardiomyocytes as a response to ischemia and acts as a local vasodilator.

Moreover, LC-MS/MS analysis demonstrates that l-glutamic acid levels were doubled for the model representing myocardial ischemia condition. Studies showing a correlation between l-glutamic acid and myocardial ischemia directly imply that cardiac cells have glutamic acid receptors, such as NMDA receptors, which are associated with ischemia.^49^ During ischemia, the cells’ uptake of oxygen and nutrients decreases, which can cause metabolic changes in the cells, which may increase the entry of l-glutamic acid into the cell and lead to stimulation of NMDA receptors. However, the particular effect and mechanisms of l-glutamic acid on cardiomyocyte ischemia are still unclear. Results from research on “detecting early myocardial ischemia in the rat heart with MALDI imaging mass spectrometry” showed that l-glutamic acid levels increased approximately 2 times compared to the control group within the first 15 min of ischemia.^50^ Although l-glutamic acid levels decreased slowly after that, they remained elevated. Since the H9C2 cells used in our study were rat cardiomyoblasts, their incorporation into our “heart-on-a-chip” system emerges as quite compatible with the literature findings.

In our chip system, myocardial ischemia was found to be associated with an increase in isoleucine levels. Numerous factors influence how isoleucine levels fluctuate; however, certain studies and clinical findings show that isoleucine levels in the blood can increase during ischemia.^51^ This may cause the ischemic heart muscle to use amino acids such as isoleucine as an energy source when required by activating protein metabolism.

Lastly, it is well-known that glutathione is an important antioxidant molecule found in cells and intercellular fluids. Under hypoxic conditions, the balance between reduced glutathione and the oxidized glutathione-disulfide (GSSG) form of glutathione may alter. Hypoxic environments may enhance the activity of antioxidant enzymes and result in reducing the ability of glutathione to balance oxidative damage.^51^ In this case, a decrease in the amount of intercellular GSSG may be observed. It was seen in this study that oxidized glutathione levels decreased with ischemia when the medium content was assessed in terms of various analytes. To detail this phenomenon, future studies are needed to determine the amount of intracellular reduced and oxidized glutathione levels together with the glutathione enzyme activity.

The obtained small-molecule data have been evaluated as correlated with the findings published by Johnson et al., where the intracellular pH decreases from 7.03 to 6.02, while intramyocardial PCO_2_ increases from 63 to 209 Torr in 4 min in rat hearts during cardiac arrest. Rather than increased CO_2_ synthesis or reduced removal, the mechanism behind this hypercarbic acidosis appeared to be the buffering of metabolic acids by bicarbonate.^52^ On the other hand, Apstein et al. revealed that the lactate generation was shown to peak in the first 10–15 min of ischemia and then decrease gradually by 40–50% in isolated rat and rabbit hearts throughout 15–60 min of subsequent ischemia.^53^ In 2005, the function of glutamic acid on cardiac function and metabolism was investigated by testing the ischemia model in cats. The anesthetized cat showed an increase in glutamate intake by the myocardium (from 9.5 to 34.7 μM), which led to an increase in anaerobic succinate synthesis when myocardial ischemia was induced.^54^

Furthermore, ADP and ATP levels were assessed during 15 min of ischemia by Jennings et al., indicating a faster decrease under in vitro conditions compared to the in vivo (canine) model.^55^ In another related study, an ischemia model was applied to the rat heart, demonstrating an increase in ADP concentration during the onset of ischemia, peaking at a 100% increase within approximately 2 min and returning to control levels after 20 min.^56^

## 4 Conclusions

In this study, we successfully developed a microchip platform that has the potential to be used as a “heart-on-a-chip” system in which cardiomyocytes embedded in a hydrogel scaffold of alginate–gelatin–collagen combination and implanted into a single-channel PDMS chip. Cardiomyocytes under ischemia were shown to produce adenosine as an adaptive reaction, causing the arterioles that nourish them to dilate. Meanwhile, carbonic acid, lactic acid, isoleucine, l-glutamic acid, adenosine, and ADP molecules, which increase in the cytoplasm of cardiomyocytes due to the change in their metabolism, emerge and enter the circulation. In our study, thanks to the controlled ischemia condition created by the chip system, these specific molecules were determined even with low media volumes, raising the possibility of elucidating disease pathogenesis at the cellular/tissue level. Considering all of these data, our chip design stands out as a promising platform for both the qualitative and quantitative detection of MI-related biomarkers with high precision, facilitating further research on the early detection of MI.

## Supporting Information Available

Hydrogel surface modification method, PDMS capillary production, additional hydrogel SEM images (PDF)
