# Supplementary material for: Hydrogel-Integrated Heart-on-a-Chip Platform for Assessment of Myocardial Ischemia Markers
Source: ACS Omega. 2024 Sep 30;9(41):42103–15. doi: 10.1021/acsomega.4c02121 (PMC11483411; doi:10.1021/acsomega.4c02121)
Supplement: Supplementary file 1 — ao4c02121_si_001.pdf [file ao4c02121_si_001.pdf]

Hydrogel-Integrated Heart-on-a-Chip Platform for Assessment of Myocardial Ischemia Markers

Berna Ates[1†], Tolga Eroglu[2†], Seray Sahsuvar[3], Ceyhun Ekrem Kirimli[1], Ozgur Kocaturk[4], Sahin Senay[5*], Ozgul Gok[1*]

1 Department of Biomedical Engineering, Faculty of Engineering and Natural Sciences, Acibadem Mehmet Ali Aydinlar University, Istanbul, Turkey; atesberna9801@gmail.com, ceyhun.kirimli@acibadem.edu.tr, ozgul.gok@acibadem.edu.tr
2 School of Medicine, Acibadem Mehmet Ali Aydinlar University, Istanbul, Turkey; tolgaeroglu00@gmail.com
3 Department of Medical Biotechnology, Institute of Health Sciences, Acibadem Mehmet Ali Aydinlar University, Istanbul, Turkey; seray.sah@gmail.com
4 Institute of Biomedical Engineering, Bogazici University, Istanbul, Turkey; ozgur.kocaturk@boun.edu.tr
5 Department of Cardiovascular Surgery, School of Medicine, Acibadem Mehmet Ali Aydinlar University, Istanbul, Turkey; sahin.senay@acibadem.edu.tr

† These authors have contributed equally to this work.
* Equal correspondence: ozgul.gok@acibadem.edu.tr; Tel.: +90 216 500 4188
sahin.senay@acibadem.edu.tr; Tel.: +90 216 500 4021

Appendices & Supplementary Figures

Appendix 1

GS1: Coating the glass slide surface with 6µL collagen for 30 min. by spin coater, then adding 200µL collagen-free Gelatin-Alginate hydrogel to the slide surface, keeping in spin coater for 15 min.;

GS2: Coating the slide surface with 6µL collagen for 15 min. by spin coater, then adding 200µL collagen-free Gelatin-Alginate hydrogel, keeping it in a spin coater for 30 min.;

GS3: Adding 200µL Gelatin-Alginate hydrogel mixed with 6µL collagen to glass slide and keeping it in a spin coater for 45 min.;

GS4 (Control slide): Adding 200µL collagen-free Gelatin-Alginate hydrogel to the glass slide and keeping it in a spin coater for 45 min.

(Spin coater parameters for each experiment= Ramp: 10.0s / Dwell: 900-2700s / rpm: 150. Concentrations of the Gelatin-Alginate solutions were the same with previous experiment steps.)

At the final step, the glass slides were exposed to water flow to test hydrogel-surface adhesion. Based on these test results, same experimental steps were repeated on the prepared PDMS surfaces for checking the collagen adhesion on glass surfaces (PDMS-s_) and PDMS capillaries (PDMS-c_). (PDMS to curing agent: 10:1 (m/m) ratio)

On the PDMS-s2 and PDMS-s3 named surfaces, GS2 and GS3 procedures were applied, respectively. (Spin coater parameters for each experiment= Ramp: 10.0s Dwell: 900-2700s rpm: 1300)

Appendix 2

PDMS Capillary Production: 5g of PDMS solution (PDMS to curing agent: 10:1 (m/m) ratio) were prepared, left in a desiccator for 5 minutes and became free of bubbles. As a result, a 5mL syringe having a tip in the center was obtained. Plunger of the syringe was removed while the needle attached to the tip of the syringe. A glass capillary tube is inserted into the injector and fit to the tip. PDMS solution was poured into the space between the injector and the glass capillary. Whole system was incubated at 80 °C for 10 minutes to cure PDMS. Afterwards, both sides of the injector were cut longitudinally with scissors and the system was immersed in a hexane bathfor 10 minutes to easily remove the injector mold around the PDMS from the glass capillary inside the PDMS. Finally, PDMS capillary was cleared of hexane by washing out with deionized water for 30 minutes, and then air-dried.

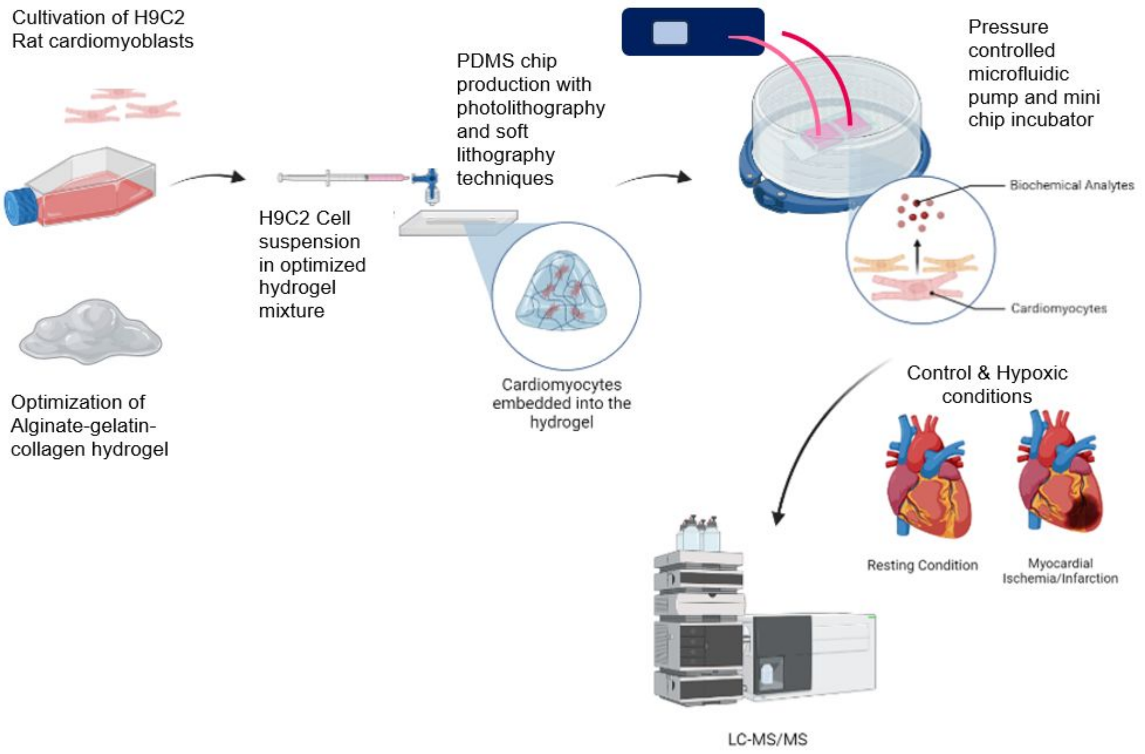

Supp. Figure 1: Workflow of the Heart-on-a-chip optimization and myocardial ischemia model. (Figure created with Biorender.com)

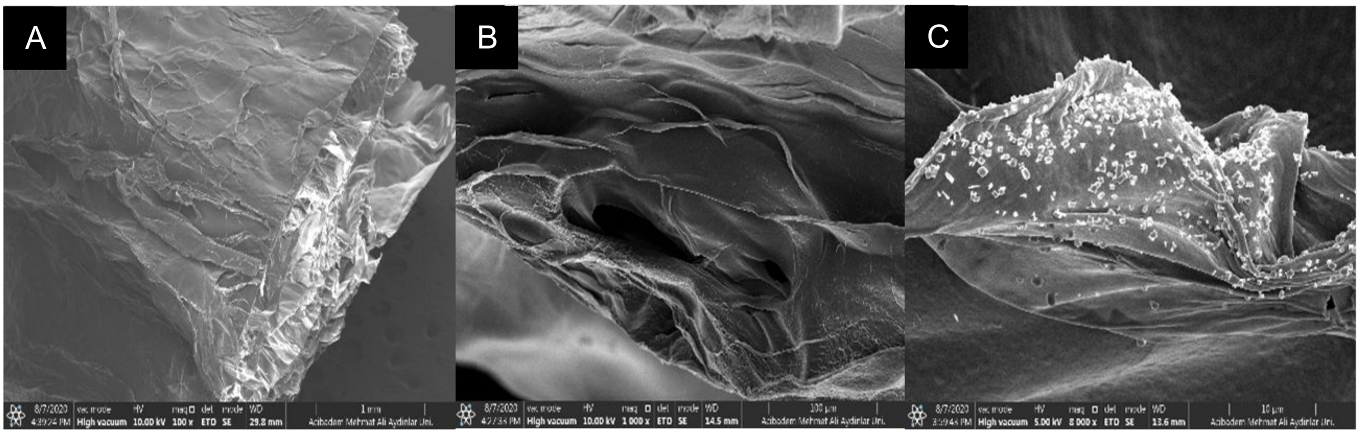

Supp. Figure 2: In image A, the quarter circle of a lyophilized hydrogel was visualized and a cut surface was examined at 100X magnification, where the separation of the porous interior and exterior surfaces is selected clearly. In Image B, reticulated structures were discovered at 1000X magnification at the surface; additionally, excess $CaPO_3$ salt crystals were observed at 8000X magnification (Image C).

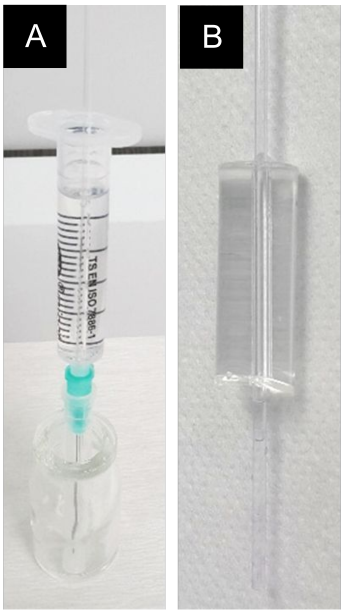

Supp. Figure 3: PDMS capillary fabrication steps.
